# Effect of Peritoneal Dialysis on Serum Fibrosis Biomarkers in Patients with Refractory Congestive Heart Failure

**DOI:** 10.3390/ijms20112610

**Published:** 2019-05-28

**Authors:** Margarita Kunin, Vered Carmon, Pazit Beckerman, Dganit Dinour

**Affiliations:** Nephrology and Hypertension Institute, Sheba Medical Center and Sackler Faculty of Medicine, Tel-Hashomer 52621, Israel; vered.carmon@sheba.health.gov.il (V.C.); pazit.beckerman@sheba.health.gov.il (P.B.); dganit.dinour@sheba.health.gov.il (D.D.)

**Keywords:** congestive heart failure, peritoneal dialysis, fibrosis markers

## Abstract

Background: Cardiac collagen remodeling is important in the progression of heart failure. Estimation of cardiac collagen turnover by serum levels of serological markers is used for monitoring cardiac tissue repair and fibrosis. Peritoneal dialysis (PD) is used for the long-term management of refractory congestive heart failure (CHF). In this study, we investigated the effect of PD treatment on circulating fibrosis markers levels in patients with refractory CHF and fluid overload. Methods: Twenty-five patients with refractory CHF treated with PD were prospectively enrolled in the study. Circulating fibrosis markers procollagen type III C-peptide (PIIINP), matrix metalloproteinase 2 (MMP-2), and tissue inhibitor of metalloproteinases I (TIMP-1) levels were checked at baseline and after three and six months of treatment. Results: The clinical benefit of PD manifested by improved NYHA functional class and reduced hospitalization rate. Serum brain natriuretic peptide (BNP) levels decreased significantly during the treatment. Serum MMP-2 and TIMP-1 decreased significantly on PD. Circulating PIIINP showed two patterns of change, either decreased or increased following PD treatment. Patients in whom circulating PIIINP decreased had significantly lower baseline serum albumin, lower baseline mean arterial blood pressure, higher serum CRP, and a less significant improvement in hospitalization rate compared to the patients in whom circulating PIIINP increased. Patients in whom all three markers decreased demonstrated a trend to longer survival compared to patients whose markers increased or did not change. Conclusion: In refractory CHF patients PD treatment was associated with a reduction in circulating fibrosis markers.

## 1. Introduction

Cardiac collagen remodeling is important in the progression of heart failure. Progressive myocardial fibrosis in response to injury is a hallmark of pathological cardiac remodeling. It involves myocyte replacement with fibrosis or diffuse deposition of extracellular matrix (ECM). The cardiac ECM consists mostly of type I collagen and type III collagen. ECM turnover is determined by the net balance of collagen synthesis and degradation [1]. During type I and type III collagen synthesis procollagen type I C-peptide (PIP) and procollagen type III C-peptide (PIIINP) release into the circulation, respectively. Degradation of extracellular matrix and deposition of collagen fibers in cardiac tissue are mainly controlled by the expression and activity of matrix metalloproteinases (MMPs) and their endogenous inhibitors. Matrix metalloproteinases are a family of zinc-dependent endopeptidases responsible for cleaving of ECM proteins [2]. MMP-2 is one of the most studied MMPs. Failing hearts display increased expression of MMP-2 and its endogenous inhibitor tissue inhibitor of metalloproteinases I (TIMP-1) at transcript- and protein- levels [3,4]. MMP-2 and TIMP-1 have been demonstrated to contribute to ventricular remodeling and myocardial apoptosis in experimental HF model [5].

Previously, collagen content in the heart was directly measured in biopsies. Recently, noninvasive methods have been developed to detect cardiac collagen content, such as circulating biomarkers of collagen synthesis and degradation. PIIINP was found to be elevated in patients with advanced heart failure and a marker of poor prognosis [6,7,8]. Circulating levels of MMP-2 and TIMP-1 are also elevated in chronic HF patients [9,10,11], significantly correlated with HF development, and correlated with the severity of heart failure [9,10,12,13,14]. Results of different clinical trials suggest that plasma MMP-2 and TIMP-1 concentrations can be useful to assess disease severity in patients suffering from hypertrophic cardiomyopathy or heart failure [13]. Plasma TIMP-1 was related to indices of LV hypertrophy and systolic dysfunction [15]. It was demonstrated that following treatment with aldosterone antagonists serum levels of PIIINP decreased [6,7,8,11]. 

Treatment of systemic congestion is one of the most difficult challenges in the management of severe congestive heart failure (CHF), particularly in patients with diuretic resistance. Peritoneal ultrafiltration (UF) is a simple choice for daily fluid removal with several advantages compared to ultrafiltration through hemodialysis: minimal impact on hemodynamics, slower decline in residual kidney function, sodium sieving with maintenance of normonatremia, possibility of draining of ascites, and avoidance of risks related to a vascular access and improvement of systemic inflammatory state [16,17]. It was demonstrated that in severe CHF patients PD improves the functional status and quality of life, reduces hospitalization rate and even may decrease mortality rate [18,19,20,21]. 

This study was designed to evaluate the net effect of peritoneal dialysis on circulating fibrosis markers levels in patients with refractory CHF and fluid overload.

## 2. Results

### 2.1. Clinical Characteristics

Twenty-five patients were enrolled into the study. The clinical, biochemical, and echocardiographic characteristics of the patients at baseline, prior to the beginning of PD, are presented in Table 1. During follow-up period six patients died. Three patients died from CHF exacerbation, two from septic foot, and one from peritonitis. Blood samples at 6-month follow-up were obtained from only 19 patients due to mortality. Complications of the procedure were peritonitis in one patient and outflow failure that needed the second surgical procedure for catheter Tenckhoff reposition in two patients.

All patients continued treatment with oral furosemide. The median dose of oral furosemide remained stable for the duration of the study and was 160 mg per day (range: 120–240 mg). Patients were instructed to add 2.5 mg of metolazone when their body weight increased. Eleven patients were treated in a CHF day care center while on PD, where they received intravenous furosemide and vasoactive agents. 

The average volume of PD solution the patients used was 3.28 ± 0.7 L per day. Fifteen patients (60%) were treated with one PD exchange per day, 7 (28%) with two exchanges, and more and 3 patients (12%) did only paracentesis. Four patients (16%) were on dialysis for 8 or more hours per day. Patients who needed only fluid removal did short PD exchanges 1–2 h each exchange. Those who had a substantial renal dysfunction were treated with longer exchanges or used cycler. At the three months of PD treatment point all patients reached a well-tolerated edema-free state. By three months of treatment body weight decreased significantly (Table 2) and the average weight loss was 8.9 kg (*p* < 0.0001). Table 2 and Table 3 present selected clinical and biochemical characteristics during patient follow-up. The clinical benefit of PD manifested by improvement of NYHA functional class and hospitalization rate (Table 2 and Table 3). Circulating CRP decreased significantly from 29.97 ± 5.9 mg/L at baseline to 12.08 ± 2.26 mg/l at 3 months (*p* = 0.0008) and 12.42 ± 3.46 mg/l after 6 months of PD (*p* = 0.0020) (Table 2 and Table 3). The 1-year survival rate was 58%. 

Fourteen patients performed a second echocardiography after PD has been started. LVEF was either stable (*n* =7), improved (*n* =3), or aggravated (*n* = 4). Estimated SPAP was either stable (*n* =6), improved (*n* =6), or aggravated (*n* = 2).

### 2.2. Circulating BNP Levels

Elevated pretreatment circulating BNP levels were found in all patients. BNP levels decreased significantly from 1564.44 ± 190.37 pg/mL to 1042.96 ± 139.64 pg/mL at 3 months (*p* = 0.0063) and 1052.55 ± 164.13 pg/mL at 6 months (*p* = 0.0110) (Table 2 and Table 3). 

### 2.3. Circulating PIIINP

Overall PIIINP levels did not significantly changed (Table 2 and Table 3), yet a subgroup of patients in whom circulating PIIINP levels decreased following PD treatment was identified. In this group (the first group) serum PIIINP decreased from 18.1 ± 2.3 micg/L at baseline to 15.1 ± 2.3 micg/L at 3 months (*p* < 0.0001), compared to the second group, where PIIINP levels increased from 14.9 ± 1.6 micg/L at baseline to 19.7 ± 2.4 micg/L (*p* = 0.0052). At 6 months of PD treatment serum PIIINP decreased from 21.6 ± 3.9 micg/L at baseline to 16.9 ± 4.6 micg/L in the first group (*p* = 0.0228), compared to the second group, where PIIINP levels increased from 15.7 ± 2.3 micg/L at baseline to 21.6 ± 2.8 micg/L (*p* = 0.0022). At 3 months of treatment, patients in whom circulating PIIINP levels decreased demonstrated lower baseline serum albumin, lower baseline mean arterial blood pressure, and higher serum CRP compared to the patients whose PIIINP levels increased (Table 4). Mean arterial blood pressure was 80.29 ± 3.43 mm Hg in the first group compared to 91.28 ± 3.71 mm Hg in the second group (*p* = 0.0438). Serum albumin was 3.15 ± 0.07 g/dL and serum CRP was 45.39 ± 11.07 mg/l in the first group compared to 3.49 ± 0.11 g/dL (*p* = 0.0171) and 12.17 ± 1.64 mg/l (*p* = 0.0057) in the second group, respectively. Hospitalization rate decreased from 22.6 ± 8.5 days to 14.1 ± 9.1 days (*p* = 0.0569) after 3 months of PD treatment in the first group compared to a decrease from 16.2 ± 5.3 days to 4.6 ± 2.7 days (*p* = 0.0096) in the second group. When diabetic and nondiabetic patients group were analyzed separately no significant changed in PIIINP levels dynamics were found between the groups at 3 and 6 months. 

### 2.4. Circulating MMP-2

MMP-2 levels decreased following PD treatment (Table 2 and Table 3) from 18.33 ± 0.92 ng/mL at baseline to 17.37 ± 0.84 ng/mL at 3 months (*p* = 0.4237), and 16.07 ± 0.8 ng/mL after 6 months on PD (*p* = 0.0285).

When diabetic and nondiabetic patients group were analyzed separately no significant changed in MMP-2 levels dynamics were found between the groups at 3 and 6 months. 

### 2.5. Circulating TIMP-1 Levels

There was a substantial decrease in serum TIMP-1 protein concentration during the treatment (Table 2 and Table 3). Serum TIMP-1 decreased from 382.38 ± 33.81 ng/mL at baseline to 332.63 ± 29.0 ng/mL at 3 months (*p* = 0.0046), and 291.71 ± 21.9 ng/mL after 6 months on PD (*p* = 0.0107). 

When diabetic and nondiabetic patients group were analyzed separately a significant decrease in serum TIMP-1 levels at 3 months was demonstrated only in the diabetic patients group. TIMP-1 levels decreased from 380.1 ± 44.9 ng/mL at baseline to 330.1 ± 41.1 ng/mL at 3 months of PD treatment (*p* = 0.0164) in diabetics compared to decrease from 387.1 ± 49.3 ng/mL to 338.0 ± 28.6 ng/mL (*p* = 0.1695) in nondiabetics. At 6 months no significant difference in TIMP-1 levels was found between diabetic and nondiabetic groups. 

### 2.6. Decrease in All Three Fibrosis Markers Correlates with Survival

**Figure 1:**
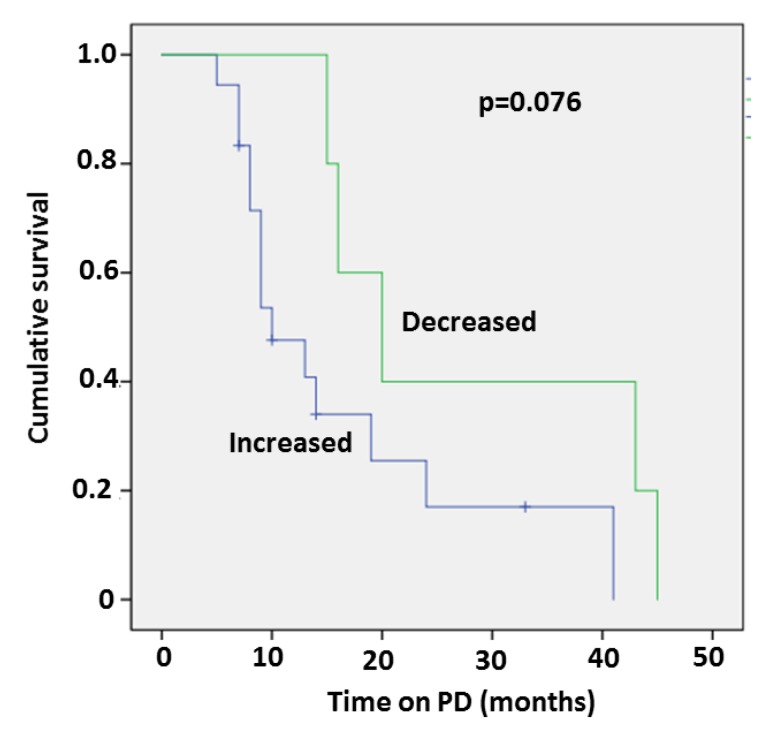
Kaplan–Meier survival curve of patients who demonstrated a decrease in all three fibrosis markers following PD treatment compared to other patients.

Five patients who demonstrated a decrease in all three fibrosis markers following PD treatment demonstrated a trend to longer survival compared to the other patients (Figure 1). In these patients, mean baseline PIIINP was higher (21.02± 2.9 micg/L) compared to the other patient group (14.55 ± 1.3 micg/L) (*p* = 0.0288).

No significant differences were found in clinical or echocardiographic parameters between patients with or without a decrease in all three fibrosis markers.

Since serum fibrosis biomarkers can be cleared by the kidney the correlation of the PIIINP, MMP2, and TIMP1 with eGFR was analyzed. Serum level of PIIINP correlated inversely with eGFR (r= −0.4961, *p* = 0.0222) while serum MMP-2 and TIMP1 did not demonstrated any correlation with eGFR (r= −0.02110, *p* = 0.9203 and r = 0.1404, *p* = 0.5033, respectively).

## 3. Discussion

Fibrosis is a common feature of various cardiomyopathies including dilated and hypertrophic cardiomyopathy and myocardial infarction [22]. The clinical presentation of cardiac fibrosis includes diastolic dysfunction, decreased pumping capacity, arrhythmias, and sudden death [23]. Serological markers that measure cardiac collagen turnover are used for monitoring cardiac tissue repair and fibrosis in experimental models or in patients [23]. 

There is only scarce data published concerning the exact timing of fibrotic deposition and degradation through the course of HF disease progression and treatment. Inter- and intrapatient differences in the activity of matrix metabolism can be observed during the evolution of HF. Guideline-directed medical therapy, in particular antagonists of the renin–angiotensin–aldosterone system, beneficially alters the course of left HF and, in selected cases, reduces fibrotic deposition [24]. Spironolactone produced a significant decrease of PIIINP serum level in patients with CHF [11,25,26]. Spironolactone effect on survival and hospitalizations was significant only in patients with above median baseline levels of PIIINP [11]. In the DOCA-salt hypertensive rat model spironolactone treatment caused improvement in functional parameters of heart failure and lower plasma MMP-2 and TIMP-1 concentrations [27]. 

In our study we observed a significant decrease in MMP-2 and TIMP-1 following PD treatment, while PIIINP decreased only in a subset of the patients. Surprisingly, PIIINP significantly decreased in a subgroup of patients in a poorer health condition, i.e., lower baseline serum albumin, lower baseline mean arterial blood pressure, and higher serum CRP. A possible explanation is that these patients with a more significant myocardial fibrosis deposition have a higher metabolic rate, and therefore the effect of PD on fibrosis could be more easily demonstrated. This is supported by the fact that this group showed a trend for higher baseline PIIINP compared to the group in which PIIINP increased or did not change. We did not see significant improvement in LVEF and SPAP following PD treatment. It was previously demonstrated that PD improves functional status, reduces hospitalization rate, decrease serum BNP levels, and may even decrease mortality rate in CHF patients [18,19,20,21]. However, in published patients groups with more advanced heart disease similar to our group echocardiographic parameters did not improved significantly during the treatment. There were fewer patients with diabetes mellitus in patients whose PIIINP significantly decreased compared to other group (50% versus 91%). It was demonstrated that among patients with HF, those with diabetes have higher mortality rates [28,29]. Studies evaluated effect of medical therapy on PIIINP level did not address diabetes mellitus patients specifically [11,25]. Spironolactone produced a significant decrease of PIIINP serum level in patients with CHF and had a positive effect on survival and hospitalizations [11]. It could be assumed that patients in the group where pIIINP was left unchanged were sicker, with a confounding effect of diabetes for worse clinical outcome. 

In our study, the 1-year survival rate was 58%; an improved survival compared to the reported 1-year survival rate of 26% for refractory HF patients under conservative treatment [30]. A decrease of all three fibrosis markers following PD treatment was associated with a trend to longer survival and higher baseline median PIIINP. This finding is in line with previous data showing a mortality benefit following spironolactone treatment in CHF patients only in patients with high (above median) baseline level of PIIINP [11]. 

Since serum MMP-2 and TIMP-1 decreased significantly and serum PIIINP did not changed significantly in the entire cohort, one could assumed that PD treatment in CHF patients has a more profound effect on collagen degradation than on collagen synthesis rate. It was found that matrix degradation rather than synthesis may be prevailed in some patients with chronic HF [11]. It was demonstrated that the circulating multimarker profile in severe symptomatic HF is consistent with enhanced fibrotic degradation compared to mild disease [31]. Guideline-directed medical therapy, in particular, antagonists of the renin–angiotensin–aldosterone system that has been shown to beneficially alter the course of left HF and in selected cases to reduce fibrotic deposition, often initiated and up-titrated during hospitalization. Thus administration of therapy that impacts fibrosis often coincides with a time of fibrotic degradation rather than synthesis during the chronic remodeling process [31]. Clinical trials of aldosterone antagonists demonstrated the importance of collagen biomarkers as a predictor of patient responsiveness to antifibrotic therapy. MMP-2 circulating levels could serve as an indicator of the efficiency of therapy in HF patients and for identification of patients who could profit from particular therapeutic intervention. However, studies showing normalization of MMP-2 serum levels by therapeutic interventions in human are lacking.

In our study TIMP-1 demonstrated the most significant decrease at both time points (3 and 6 months) following PD treatment. A rise in TIMP-1 promotes fibrosis by inhibiting MMPs and their ECM-degrading function [32,33]. A novel, MMP-independent mechanism for TIMP1 action was recently described [34]. TIMP1 was shown to induce myocardial fibrosis through mediating an interaction between fibroblast membrane proteins, CD63, and integrin β1 [34]. The authors assumed that targeting TIMP-1 could provide a new direction into developing antifibrosis therapies [34]. 

Angiotensin II stimulates collagen synthesis and regulates collagen degradation by enhancing TIMP-1 production in endothelial cells, thereby attenuating interstitial MMP-1 activity [35,36]. Chronic AT1 blockade with losartan resulted in inhibition of TIMP-1 expression and stimulation of collagenase activity in the left ventricle of spontaneously hypertensive rats [37]. Fluid removal during PD treatment resetting neurohumoral activation towards a more physiological state may be responsible for our observed decrease in TIMP-1.

We assume that in some refractory CHF patients with fluid overload, PD treatment can lower the circulating levels of fibrosis markers adding benefit to already maximally tolerated traditional drug regimens. The possible explanations for PD benefits in cardiac fibrosis are neurohumoral activation resetting towards a more physiological condition after fluid removal, the decrease in mechanical pressure on the heart resulting in less remodeling, improvement in inflammation, and perhaps also the decrease in uremic toxins levels. All those factors are found to contribute to cardiac fibrosis [38,39]. We do not think that removal of PIIINP, TIMP-1, and MMP-2 by the peritoneal membrane had a significant impact on fibrosis markers plasma levels. Most of our patients perform one or two short dialysis exchanges per day. PIIINP, TIMP-1, and MMP-2 have a high molecular weight of 42 kDa, 28 kDa, and 72 kDa, respectively; therefore their removal by PD program including a few short exchanges per day is unlikely. 

The limitations of the study include the small number of patients and their heterogeneity. An important question is, is the serum biomarker representative at the tissue level? Our analysis assumes that biomarkers are representatively excreted into circulation and that they actually reflect the myocardial accumulation of fibrous tissue. Further studies are needed to correlate the serum levels of the biomarkers with histology of cardiac biopsies and to define the precise timing of fibrotic deposition and degradation through the course of HF disease progression and treatment; this may facilitate temporal targeting of antifibrotic therapies and a better utilization of PD in this high risk population. Compared to cardiac biopsy, which is an invasive procedure with a risk of such complications as myocardial perforation with pericardial tamponade, arrhythmias, and pneumothorax [40], serum biomarkers represent a simple, noninvasive tool that could be used repeatedly for follow-up and, if confirmed to be reliable, could replace cardiac biopsies in evaluation of patients with heart failure. 

## 4. Materials and Methods

### 4.1. Subjects 

All procedures performed in studies involving human participants were in accordance with the ethical standards of the institutional and/or national research committee and with the 1964 Helsinki declaration and its later amendments or comparable ethical standards. 

Patients with refractory CHF referred by their cardiologists to our PD unit between March 2012 and July 2016 and completed at least a 3-month period of PD were enrolled into this study. The study protocol was approved by the Sheba Medical Center Institutional Human Research Board (19 January 2010). Written informed consent was obtained from all participants.

All patients were in NYHA functional class IIIb or IV with symptoms and signs of severe cardiac failure and volume excess. They received optimal medical therapy according to the Heart Failure Guidelines [41]. This included dietary fluid and salt restriction and maximal tolerable medication therapy, including diuretics—loop and distal tubule (metolazone)—angiotensin-converting enzyme inhibitor (ACEI) or angiotensin II receptor blockers (ARB), beta-blockers, and digoxin. Some of the patients were also treated with intravenous furosemide and vasoactive agents in a CHF day care center. Inclusion criteria for PD: (1) NYHA functional class IIIb or IV; (2) an echocardiographic evidence of significant left or right ventricular dysfunction, valvular heart disease or pulmonary hypertension; and (3) significant volume overload despite maximal doses of diuretics or repeated episodes of deteriorating kidney function (defined as a 50% increase in serum creatinine from basal concentration) during intensification of diuretics treatment or recurrent hospitalizations for volume overload in the preceding 3 months. Patients with some relative contraindication for PD (such as severe lung disease, extensive abdominal scars, and abdominal aortic aneurism) or inability to comply with the procedure of PD, were excluded from the study. 

### 4.2. Peritoneal Dialysis Description

A PD catheter was implanted under local anesthesia by surgical dissection in the operating room. In patients with ascites, peritoneal centesis was started by a trained PD nurse one day after Tenckhoff catheter insertion. In patients with a significant volume overload, volume exchanges (~1500 mL) were performed in the recumbent position by a PD nurse starting the day after catheter placement. Dwell times were 1–2 h for one to three exchanges per day, 2–4 days per week. PD exchanges were performed by a PD nurse in the PD unit until training of the patient or a family member was satisfactory (approximately 2–3 weeks). 

PD solutions included both glucose- and nonglucose-containing (icodextrin) dialysis solutions (Teva Medical, Israel, and Cure Medical and Technical Supply, Fresenius, Germany). Data on the type and volume of PD solutions used and daily peritoneal UF volume were gathered.

### 4.3. Clinical Evaluation 

The following clinical parameters were collected; disease etiology, functional status (NYHA), preserved/reduced LV function, comorbidities and medications, body weight, and mean arterial blood pressure. Assessment of fluid status was based on clinical examination. The manifestations of volume overload were considered pulmonary congestion, peripheral edema, and elevated jugular venous pressure. Laboratory results included serum hemoglobin and leukocyte count, serum albumin, sodium, urea, creatinine, uric acid, erythrocyte sedimentation rate (ESR), and C-reactive protein (CRP). Estimated GFR (eGFR) was calculated using the Modification of Diet in Renal.

Disease formula: Primary kidney disease was defined as urine protein >0.5 g/24 h, abnormal urine microscopy, and/or abnormal renal sonography (e.g., unequal kidney size or reduced thickness of renal parenchyma). Patients with urine protein <0.5 g/24 h, normal urine microscopy, and normal kidneys per sonography were classified as having cardiorenal syndrome. Echocardiographic parameters used in the study included LVEF and RVEF and systolic pulmonary artery pressure (SPAP). 

Peripheral venous blood samples were collected and immediately centrifuged, and the serum samples were stored at −80°C until assay. Serum was collected from the patients at baseline, i.e., before the first PD treatment, and at 3 and 6 months of therapy, when available. 

### 4.4. BNP Assay 

BNP was measured using the Alere Triage^®^ BNP Test, a rapid fluorescence immunoassay kit (Quidel, San Diego, CA, USA).

### 4.5. Matrix Metalloproteinases ELISA Assays 

Plasma levels of MMP-2 and TIMP-1 were assessed by enzyme-linked immunosorbent assay (ELISA) as per the manufacturer’s protocol (R&D Systems, Minneapolis, MN, USA). This assay employs the quantitative sandwich enzyme immunoassay technique. The cut-off or lower limit of sensitivity ranges from 0.014 to 0.082 ng/mL for MMP-2 and less than 0.08 ng/mL for TIMP-1. 

### 4.6. Procollagen-III-Peptide ELISA Assay

Plasma PIIINP level was assessed by ELISA assay per manufacturer protocol (Cisbio, Coolest, France). This assay employs one-step sandwich colorimetric ELISA-type immunoassay. The cut-off or lower limit of sensitivity is 0.036 micg/L.

### 4.7. Statistical Analysis 

Statistical analyses were performed by GraphPad InStat 3 (GraphPad Software, San Diego, California, USA). Data are presented as mean ± SEM for continuous variables and as absolute numbers and percentages for categorical variables. Data presented in Table 2 and Table 3 were compared by two-tailed paired Student’s *t*-test. Data presented in Table 4 were compared by two-tailed Student’s *t*-test for quantitative variables. Categorical variables were analyzed by Fisher’s exact test. Survival was assessed by Kaplan–Meier survival analysis. Pearson’s correlation test was used to analyze the association between circulating fibrosis markers levels and eGFR. *p* < 0.05 was considered significant. 

## 5. Conclusions

This study measured for the first time circulating fibrosis markers in severe congestive heart failure patients treated with peritoneal dialysis for fluid overload. A decrease in the serum fibrosis markers following PD treatment was demonstrated. Reduction in circulating fibrosis markers levels was associated with a trend to longer survival. This demonstrated that PD is a beneficial treatment, not just symptomatic but may increases survival and it is maybe also through its effect on cardiac fibrosis.

## Figures and Tables

**Table 1:** Selected clinical, biochemical, and echocardiographic characteristics of the patients at baseline.

Variables		Mean
Age (years)		66.4 ± 1.79
Females		4 (16%)
Ischemic cardiomyopathy		16 (64%)
NYHA class III/IV		8/17
Diabetes mellitus		17 (68%)
History of hypertension		17 (68%)
Primary kidney disease		11 (44%)
Body weight (kg)		86.11 ± 3.26
MAP (mm Hg)		84.46 ± 2.44
eGFR (mL/min/1.73 m²)		32.5 ± 3.5
LVEF (%)		31.7 ± 4
Preserved LV function		8 (32%)
RV dysfunction		14 (56%)
Estimated SPAP (mm Hg)		60.13 ± 3.77
CHF day care treatment		11 (44%)
Medications	Loop diuretic	25 (100%)
	Thiazide and thiazide-like diuretics, metolazone	6 (24%)
	Spironolactone	9 (36%)
	Beta-blockers	24 (96%)
	Digoxin	10 (40%)
	ACEI or ARB	8 (32%)

Values expressed as mean ± SEM for continuous variables and as absolute numbers and percentages for categorical variables. eGFR—estimated Glomerular Filtration Rate; MAP - Mean Arterial Pressure; LVEF—Left Ventricular Ejection Fraction; RV—Right Ventricle; SPAP—Systolic Pulmonary Artery Pressure; ACEI—Angiotensin-Converting-Enzyme Inhibitor; ARB—Angiotensin II Receptor Blocker .

**Table 2:** Selected clinical and biochemical characteristics after 3 months of PD treatment (*n* = 25).

Variables	Baseline	3 Months	*p*-value
Body weight (kg)	86.12 ± 3.26	77.21 ± 3.08	<0.0001
NYHA class IV (%)	17 (68%)	6 (24%)	0.0041
Hospitalization (day/month)	17.3 ± 4.2	8.8 ± 3.9	0.0012
Residual renal volume (L)	1.25 ± 0.14	1.24 ± 0.13	0.8921
Serum creatinine (mg/dL)	2.53 ± 0.22	2.42 ± 0.25	0.4556
eGFR (mL/min/1.73 m²)	32.5 ± 3.5	35.64 ± 3.61	0.1471
Serum urea (mg/dL)	174.68 ± 12.66	119.96 ± 6.5	<0.0001
Serum sodium (mEq/L)	135.56 ± 0.77	134.88 ± 0.83	0.3749
Serum uric acid (mg/dL)	10.6 ± 0.55	8.8 ± 0.42	0.0063
Serum albumin (g/dL)	3.4 ± 0.08	3.02 ± 0.09	0.0007
Serum hematocrit (%)	32.83 ± 1.15	35.99 ± 1.12	0.0011
Serum WBC (1000/μL)	6.5 ± 0.43	7.45 ± 0.42	0.0285
Serum CRP (mg/L)	29.97 ± 5.9	12.08 ± 2.26	0.0008
Serum BNP (pg/mL)	1564.44 ± 190.37	1042.96 ± 139.64	0.0063
Serum PIIINP (micg/L)	16.4 ± 1.37	17.5 ± 1.72	0.3400
Serum MMP-2 (ng/mL)	18.33 ± 0.92	17.37 ± 0.84	0.4237
Serum TIMP-1 (ng/mL)	382.38 ± 33.81	332.63 ± 29.0	0.0046

Values expressed as mean ± SEM. eGFR—estimated Glomerular Filtration Rate; WBC—White Blood Cells; CRP—C-reactive Protein; BNP—Brain Natriuretic Peptide; PIIINP—Procollagen type III C-peptide; MMP-2—Matrix Metalloproteinase 2; TIMP-1—Tissue Inhibitor of Metalloproteinases I .

**Table 3:** Selected clinical and biochemical characteristics after 6 months of PD treatment (*n* = 19).

Variables	Baseline	6 Months	*p* value
Body weight (kg)	89.65 ± 3.69	79.67 ± 3.39	<0.0001
NYHA class IV (%)	12 (60%)	4 (20%)	0.0225
Hospitalization (day/month)	26.4 ± 8.0	16.8 ± 5.5	0.0754
Residual renal volume (L)	1.29 ± 0.14	1.15 ± 0.12	0.4663
Serum creatinine (mg/dL)	2.53 ± 0.22	2.81 ± 0.4	0.2977
eGFR (mL/min/1.73 m²)	32.5 ± 3.5	34.12 ± 4.66	0.6571
Serum urea (mg/dL)	159.45 ± 13.08	121.75 ± 8.15	0.0031
Serum sodium (mEq/L)	135.9 ± 0.83	136.4 ± 0.7	0.6089
Serum uric acid (mg/dL)	10.65 ± 0.57	8.78 ± 0.4	0.0217
Serum albumin (g/dL)	3.4 ± 0.09	3.25 ± 0.08	0.1465
Serum hematocrit (%)	32.29 ± 1.39	34.62 ± 1.0	0.0071
Serum WBC (1000/μL)	6.66 ± 0.48	7.54 ± 0.39	0.0940
Serum CRP (mg/L)	29.67 ± 6.94	12.42 ± 3.46	0.0020
Serum BNP (pg/mL)	1500.35 ± 185.67	1052.55 ± 164.13	0.0110
Serum PIIINP (micg/L)	17.13 ± 2.05	20.40 ± 2.35	0.0809
Serum MMP-2 (ng/mL)	18.94 ± 1.06	16.07 ± 0.8	0.0285
Serum TIMP-1 (ng/mL)	360.45 ± 25.35	291.71 ± 21.9	0.0107

Values expressed as mean ± SEM. eGFR—estimated Glomerular Filtration Rate; WBC—White Blood Cells; CRP—C-reactive Protein; BNP—Brain Natriuretic Peptide; PIIINP—Procollagen type III C-peptide; MMP-2—Matrix Metalloproteinase 2; TIMP-1—Tissue Inhibitor of Metalloproteinases I.

**Table 4:** Comparison of selected clinical and laboratory parameters of congestive heart failure (CHF) patients whose serum PIIINP decreased after 3 months of PD treatment with parameters of patients whose serum PIIINP increased or did not change (*n* = 21).

Variables	Decreased (*n* = 10)	Increased or Unchanged (*n* = 11)	*p*-Value
Age (years)	66.4 ± 2.76	63.55 ± 2.58	0.4590
Sex (f/m)	2/8	2/9	1.0000
CMP (ischemic/nonischemic)	7/3	7/4	1.0000
NYHA class III/IV	2/8	4/7	0.6351
Diabetes mellitus	5 (50%)	10 (91%)	0.0635
History of hypertension	8 (80%)	7 (64%)	0.6351
Primary kidney disease	2 (20%)	7 (64%)	0.0805
MAP (mm Hg)	80.29 ± 3.43	91.28 ± 3.71	0.0438
LVEF (%)	28.7 ± 7.5	30.9 ± 5.3	0.8086
Preserved LV function	3 (30%)	3 (27%)	1.0000
RV dysfunction	6 (60%)	8 (73%)	0.6594
LVH	2 (20%)	2 (18%)	1.0000
Estimated SPAP (mm Hg)	60.6 ± 5.29	59 ± 5.25	0.8326
LV diastolic dimention (cm)	5.89 ± 0.6	5.67 ± 0.4	0.7585
LV systolic dimention (cm)	4.7 ± 0.64	4.65 ± 0.48	0.9543
Estimated LV mass index (g/m^2^)	131.44 ± 22.11	136.64 ± 18.74	0.8589
LA diameter (cm)	5.02 ± 0.27	4.74 ± 0.21	0.4015
Serum creatinine (mg/dL)	2.41 ± 0.43	2.67 ± 0.28	0.6085
Serum albumin (g/dL)	3.15 ± 0.07	3.49 ± 0.11	0.0171
Serum sodium (meq/L)	134.5 ± 1.62	136.18 ± 0.84	0.3557
Serum CRP (mg/L)	45.39 ± 11.07	12.17 ± 1.64	0.0057
Serum BNP (pg/mL)	1700.9 ± 329.91	1767.01 ± 267.51	0.8768
Survival (m)	18.3 ± 4.43	17.55 ± 3.37	0.8923

Values expressed as mean ± SEM for continuous variables and as absolute numbers and percentages for categorical variables. CMP—cardiomyopathy; MAP - Mean Arterial Pressure; LVEF—Left Ventricular Ejection Fraction; RV—Right Ventricle; SPAP—Systolic Pulmonary Artery Pressure; LVH—Left Ventricular Hypertrophy; LA—Left Atrium; CRP—C-reactive Protein; BNP—Brain Natriuretic Peptide.
